# Knowledge about methods of disinfection of dental impression among dentists from Ecuador post SARS-COV-2 pandemic

**DOI:** 10.1016/j.heliyon.2023.e23280

**Published:** 2023-12-05

**Authors:** Myriam Lagla Abata, Gabriela Balarezo Lasluisa, María Rodriguez Tates, Byron Velásquez Ron

**Affiliations:** a Specialist in Oral Rehabilitation, Department Prosthesis Research School of Dentistry, UDLA Quito Ecuador; b Professor Department Prosthesis Research School of Dentistry, UDLA Quito Ecuador; c Dentistry Resident, Faculty of Dentistry. University of Las Americas. UDLA. Campus Colón. +593958916317 CP 170523 Quito-Ecuador, Ecuador; d Department Prosthesis Research School of Dentistry, Universidad de Las Americas (UDLA), Av. Colón y 6. Diciembre CP 170523, + 593023981000 ext 3060,Quito, Ecuador

**Keywords:** Dentistry, COVID-19, Disinfection, Glutaraldehyde, Sodium hypochlorite, Dental impingement materials

## Abstract

**Purpose:**

The aim of this study is to evaluate the understanding of the management of methods and practices of disinfection of dental impressions applied by general dentists and specialists in oral rehabilitation, post SARS-COV-2 pandemic.

**Introduction:**

The oral cavity has a favorable environment for the growth and multiplication of bacteria and viruses, serving as the gateway to viruses such as SARS CoV-2 virus. Given that patients may be hosts of this infectious disease, stricter biosecurity measures in dental offices and a better understanding of the disinfection processes of dental impressions should be considered in addition to avoiding cross-infections, which are very common in our work environment.

**Materials and methods:**

Descriptive, analytical, survey with the topic "Methods and practices of disinfection of dental impressions" that was composed of 14 questions based in OHPD (Oral Health Preventive Dentistry), n = 452 respondents who met the following inclusion criteria: general practice dentists from Ecuador who actively take dental impressions, specialists in oral rehabilitation with 5 years of experience, surveys with information of complete items, n = 270 professionals; n = 192 general dentists, dental hygienist and n = 78 oral rehabilitation specialists.

**Results:**

n = 270 professionals evaluated, with a mean of p = 0.0. At 426 responses with a p value of <0.05, 30.4 % of responses indicated ignorance in the management of dental impressions, with a standard deviation of 2.744, with significant differences between professions (p = 0.035) and higher average knowledge of sample management within specialist dentists, (Chi p = 0.410 p > 0.05)

**Conclusion:**

The understanding of the management of dental impressions among general dentists and specialists in oral rehabilitation is limited.

## 1 Introduction

After the COVID-19 pandemic, dental work in dental offices became complex [1]. The permissible spaces for the dispersion of contagious and lethal diseases between patients and dental staff has increased, new protocols and biosecurity measures were established in dental practice [2], and routine procedures such as taking dental impressions require greater care because the oral cavity is a culture medium [3] of diverse microbial flora. ,In addition, having a humid medium with adequate temperature (37 °C) promotes an optimal environment for cultivation or spread of bacteria, viruses, and spores.

Dental printing without disinfection can become a vehicle for cross-infection in the International Dental Federation (FDI), American Dental Association (ADA) and British Dental Association (BDA) note that dental impressions must be disinfected before casting or being transferred to the laboratory using universal post pandemic protocols recommended by the WHO [3,4]. Dental impressions, instruments and dental equipment are exposed to secretions and aerosols from the oral cavity (turbine water during dental surgery, saliva, blood, exudate) [5,6], therefore requiring disinfection protocols to counteract the activity of viruses, bacteria, fungi, spores, yeasts, etc. Microbes can even be found in the plaster model, resulting in transport of these microorganisms to dental laboratories at the time of trimming and handling [7]. The antimicrobial property of chemical disinfectants was assessed by measuring microbial counts in soybean trypticase agar (TSA) media [8]. The dimensional stability of printing materials following immersion in disinfectants was assessed by measuring the linear displacement of horizontally restricted materials using a movable microscope. Alginate exhibited a higher microbial balance than silicone [9]. Chemical disinfection with glutaraldehyde-based disinfectant was powerful in eliminating all microbial forms for both alginate and silicone without varying dimensional stability. The responsibility of dental professionals [9] when taking biosecurity measures for cross-infection control is higher post pandemic. Through pilot testing in a questionnaire of 10 random questions to 130 dentists of private clinics, 85 % did not disinfect the impressions before sending it to the laboratory, concluding that cross infection control measures [10] should be improved in private dental practice [11,12]. The management of biosecurity even more post pandemic must be considered properly, the preparation of this study allowed to verify that the professionals have relaxed with respect to the precautionary measures, the apprehension of digital impressions limit the cross contamination, unfortunately in the country the access to these technological tools is still limited, for this reason we raise to the scientific community this reminder for all our professional guild. The objective of the study was to evaluate the understanding of the management of methods and practices of disinfection of dental impressions applied by general dentists and oral rehabilitators post SARS-COV-2 pandemic.

## 2 Materials and Methods

This study was a descriptive, analytical, survey with the topic "Methods and practices of disinfection of dental impressions" and composed of 14 questions based in OHPD and n = 452 respondents who met the following inclusion criteria: general practice dentists from Ecuador (Quito, Guayaquil and Cuenca city's); database provided by the Ecuadorian Dental Federation, who provided e-mail address; names and surnames of the dentists surveyed and their specialty; selecting general practice dentists and specialists in oral rehabilitation with 5 years of experience, surveys with information of complete items. With the data collected, the questionnaire is elaborated in Survey Monkey, indicating the first phase the desire to participate or not in the study, validated the questions by 2 external experts, the questionnaire is sent to the registered electronic addresses, excluding those emails that were with error; The questionnaire was designed so that dentists and specialists the moment of accepting to make the questionnaire were displayed the questions. The calculation of the sample is done with the application Survey Monkey,(Menlo Park/California, USA, version 5). The sample size formula was applied:

$$\frac{\frac{z^{2}\times p\left(1-p\right)}{e^{2}}}{1+\frac{z^{2}\times p\left(1-p\right)}{e^{2}N}}$$

Population Size = 462.

Confidence Level = 95 %

Margin of error = 5 %

Suggested sample n = 217.

After the follow-up to the surveys, the sample that met the inclusion criteria were n = 270 professionals; n = 192 general dentists and n = 78 specialists in oral rehabilitation (Table 1).The questionnaire OHPD consisted of 14 closed questions, 3 ″Yes or No" questions to assess the professionals’ knowledge on disinfection of dental impressions, and 11 multiple choice questions on the methods and substances used. The survey addressed the following topics: disinfection of prints; type of disinfectant solution used for alginates and elastomeric printing materials; the time required for the disinfection of printing materials; and the disinfection technique (spraying, immersion) used for alginates and elastomeric printing materials. Through the free software application Survey Monkey,(Menlo Park/California, USA, version 5)., we proceeded to edit the questions. (Table 2).

**Table 1:**
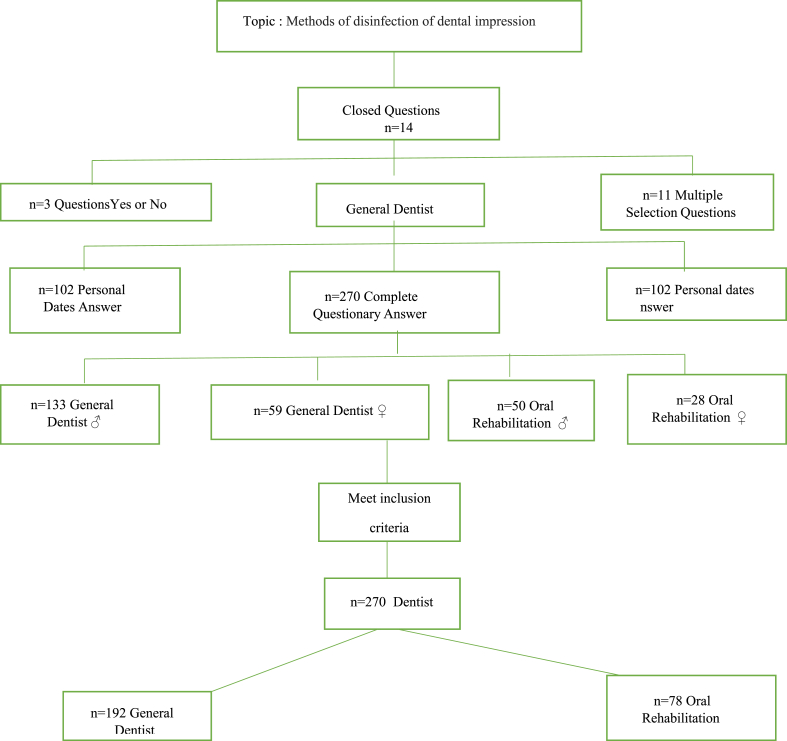
Flow Diagram research.

**Table 2:** Questions OHPD.

	Questions
1	Are you aware of the disinfection protocol for dental impressions?
2	Have you been told how to disinfect dental impressions?
3	Do you disinfect dental impressions?
4	What agent do you use to disinfect dental impressions taken with alginate and how do you use it?
5	How and for how long do you disinfect dental impressions taken with alginate?
6	What disinfectant agent do you use to disinfect dental impressions taken with Silicone Addition and how do you use it?
7	How long do you disinfect dental impressions taken with Silicone Addition?
8	What disinfectant agent do you use to disinfect dental impressions taken with Silicone Addition and how do you use it?
9	How long do you leave dental impressions taken with Condensation Silicone in disinfection solution?
10	What disinfectant agent do you use to disinfect dental impressions taken with mercaptan and how do you use it?
11	How long do you disinfect dental impressions taken in mercaptan?
12	What disinfectant agent do you use to disinfect dental impressions taken with zinquenolica paste?
13	How long do you disinfect dental impressions taken with zinquenolica paste?
14	How do you transport the disinfected dental impression or diagnostic model to the laboratory?

Statistical Descriptive analysis, total of 452 dentists joined the system with 102 provided personal data, 270 completed the questionnaire and 80 did not answer the questionnaire.

With respect to the academic degree, a data from 192 general practice dentists and 78 specialist, 50 female oral rehabilitators and 28 men were obtained.

This study was a descriptive, analytical, survey with the topic "Methods and practices of disinfection of dental impressions" and composed of 14 questions based in OHPD and n = 452 respondents who met the following inclusion criteria: general practice dentists from Ecuador (Quito, Guayaquil and Cuenca city's) who actively take dental impressions, specialists in oral rehabilitation with 5 years of experience, surveys with information of complete items. After the follow-up to the surveys, the sample that met the inclusion criteria were n = 270 professionals; n = 192 general dentists and n = 78 specialists in oral rehabilitation.

The questionnaire OHPD consisted of 14 closed questions, 3 ″Yes or No" questions to assess the professionals’ knowledge on disinfection of dental impressions, and 11 multiple choice questions on the methods and substances used. The survey addressed the following topics: disinfection of prints; type of disinfectant solution used for alginates and elastomeric printing materials; the time required for the disinfection of printing materials; and the disinfection technique (spraying, immersion) used for alginates and elastomeric printing materials. Through the free software application "Lime Survey", we proceeded to edit the questions. They are sent exclusively to the email address registered in the database of the Ecuadorian Dental Federation. (Table 2).

## 3 Results

The Chi Square test was used, comparing the sample collected randomly, with a single categorical variable. In question 1, 67.7 % of general dentists and 80 % of oral rehabilitation specialists answered yes; in question 2, 67.7 % and 70.5 %, respectively, answered yes; in question 3, 70.8 % and 84.6 % answered yes, with significant differences at Chi P > 0.005, between academic degrees (Table 3).

**Table 3:** Questions 1, 2, and 3 yes or No answers.

		General Dentistry		Oral Rehabilitation		Total		Chi (p = )
Questions	Answer	n =	%	n =	%	n =	%	
1	YES	130	67.7	63	80.8	193	71.5	0.031
	NO	62	32.3	15	19.2	77	28.5	
2	YES	116	60.4	55	70.5	171	63	0.119
	NO	76	39.6	23	29.5	99		
3	YES	136	70.8	66	84.6	202	74.8	0.018
	NO	56	29.2	12	15.4	68	25.2	

In question 4, 31.13 % of general dentists and 33.3 % of oral rehabilitation specialists reported disinfecting by spraying hypochlorite; in question 5, 33.9 % and 39.7 % of respective groups reported disinfecting by wrapping it in napkins soaked with 1 % hypochlorite packaged in an airtight bag; in questions 6 and 8, 34,4 % and 36,6 % reported immersing it in hypochlorite or glutaraldehyde; in question 10, 59.4 % and 56.4 % did not know or did not answer; and in question 12, 55.7 % and 53.8 % did not know or did not answer. There were no significant differences between academic degrees Chi (P > 0.05) (Table 4).

**Table 4:** Disinfectant agent used (questions 4, 5, 6, 8, 10, and 12).

		General Dentistry		Oral Rehabilitation		Total		Chi (p = )
Question	Answer	n =	%	n =	%	n =	%	
4	Hypochlorite	60	31.3	26	33.3	86	31.9	0.441
	Iodoform	4	2.1	3	3.8	7	2.6	
	Glutaraldehyde	53	27.6	24	30.8	77	28.5	
	Do not know/no answer	37	19.3	8	10.3	45	16.7	
**Alginate**								
5	Hermetic bag	18	9.4	10	12.8	28	10.4	0.627
	Soaked napkin	50	26	19	24.4	69	25.6	
	Hermetic bag and soaked napkin	65	33.9	31	39.7	96	35.6	
	Napkin with iodoform	10	5.2	4	5.1	14	5.2	
	Do not know/no answer	49	25.5	14	17.9	63	23.3	
**Addition**								
6	Rocio with Iodoform/Glutaraldehyde	25	13	10	12.8	35	23	0.031
	Dip with Iodoform/Glutaraldehyde	24	12.5	21	26.9	45	16.7	
	Dip Hypochlorite/Glutaraldehyde	66	34.4	27	34.6	93	34.4	
	Dip Hypochlorite/Iodoform	20	10.4	7	9	27	4	
	Do not know not answer	57	29.7	13	16.7	79	25.9	
**Condensation**								
8	Spray with Iodoform/Glutaraldehyde	27	14	11	14.1	38	14.1	
	Dip with Iodoform/Glutaraldehyde	23	12	15	19.2	38	14.1	
	Dip Hypochlorite/Glutaraldehyde	54	28	28	35.9	82	30.4	
	Dip Hypochlorite/Iodoform	26	13.5	8	10.3	34	12.6	
	Do not know/no answer	62	32.3	16	20.5	78	28.6	
**Mercaptanos**								
10	Spray with Iodoform/Glutaraldehyde	24	12.5	6	7.7	30	11.1	0.653
	Dip with Iodoform/Glutaraldehyde	5	2.6	3	3.8	8	3	
	Dip Hypochlorite/Glutaraldehyde	24	12.5	13	16.7	37	13.7	
	Dip Hypochlorite/Iodoform	25	13	12	15.4	37	13.7	
	Do not know/no answer	114	59.4	44	56.4	158	58.8	
12	**Pasta Zinquenolica**							
	Spray with Iodoform/Glutaraldehyde	12	6.3	2	2.6	14	5.2	0.481
	Dip with Iodoform/Glutaraldehyde	32	16.7	19	24.4	51	18.9	
	Dip Hypocholorite/Glutaraldehyde	32	16.7	11	14.1	43	15.9	
	Dip Hypocholorite/Iodoform	9	4.7	4	5.1	13	4.8	
	Do not know/no answer	107	55.7	42	53.8	149	55.2	

In question 7, 41.7 % of general dentists and 50 % of specialists in oral rehabilitation answered “10 min”, with a significant difference of p > 0.05 between academic degrees; in question 9, 41.1 % and 47.4 % of the respective groups answered “10 min”; in question 11, 60.9 % and 53.8 % did not know or did not answer; In question 13, 57.3 % and 52.6 % answer did not know or did not answer. There were no significant differences between academic degrees (p > 0.05) (Table 5).

**Table 5:** Disinfection time (Questions 7, 9, 11, and 13).

		General Dentistry		Oral Rehabilitation		Total		Chi (p = )
Question	Answer	n =	%	n =	%	n =	%	
7	1 min	41	21.4	20	25.6	61	22.6	0.042
	10 min	80	41.7	39	50	119	44.1	
	30 min	17	27.6	24	30.8	77	28.5	
	1 h	1	8.9	4	5.1	21	7.8	
	Do not know/no answer	53	27.6	12	15.4	65	24.1	
**Condensation**								
9	1 min	35	18.2	19	24.4	54	20	0.140
	10 min	79	41.1	37	47.4	116	43	
	30 min	17	8.9	5	6.4	22	8.1	
	1 h	1	0.5	2	2.6	3	1,1	
	Do not know/no answer	60	31.3	15	19.2	75	27.8	
**Mercaptanos**								
11	1 min	19	9,9	8	10.3	27	10	0.529
	10 min	38	19.8	15	19.2	53	19.6	
	30 min	16	8.3	12	15.4	28	10.4	
	1 h	2	1	1	1.3	3	1.1	
	Do not know/no answer	117	60.9	42	53.8	159	58.9	
**Zinquenolica Pastes**								
13	1 min	50	26	26	33.3	76	28.1	0.788
	10 min	17	8.9	5	6,4	22	8.1	
	30 min	13	6.8	5	6.4	18	6.7	
	1 h	2	1	1	1.3	3	1.1	
	Do not know/no answer	110	57.3	41	52.6	151	55.9	

In question 14, 80.2 % of general dentists and 89.7 % of specialists in oral rehabilitation, answered “in hermetic bags” and there were no significant differences between academic degrees Chi (p > 0.05) (Table 6).

**Table 6:** Transportation to laboratory.

		General Dentistry		Oral Rehabilitation		Total		Chi Test (p = )
Question	Answer	n =	%	n =	%	n =	%	
14	On the hand	4	2.1	0	0	4	1.5	0.348
	Hermetic bag Bolsa Airtight	154	80.2	70	89.7	224	83	
	On the Napkin	12	6.3	2	2,6	14	5.2	
	None	4	2.1	1	1.3	5	1.9	
	Do not know/no answer	18	9.4	5	6.4	23	8.5	

## 4 Discussion

Dental procedures, without biosecurity measures and sample handling protocols that consider the handling of dental impressions and their transport, can cause infections into the bloodstream [13,14]. Educating dentists about infection control can minimize the chances of cross-infection transmission, especially during the SARS-COV-2 pandemic [15,16].

The route of cross-infection begins with dental impressions, which will drag a series of microorganisms (bacteria, fungi, viruses) present in oral tissues, saliva, blood [17,18], tartar which can become opportunistic pathogens in susceptible subjects. These germs can remain viable after emptying for at least 24 h [19].

The most commonly used disinfectants are sodium hypochlorite, glutaraldehyde, iodine and potassium peroxysulfate. The U.S. Environmental Protection Agency maintains an updateor list of antibacterial products based on their specific effects [20,21]. From it we highlight some active ingredients with which we are accustomed to working in the clinic and that have demonstrated efficacy against Mycobacterium Tuberculosis (TB), HIV and HBV; such as 0.5 % hydrogen peroxide, 0.6 % citric acid, 0.55 % sodium hypochlorite, 70 % isopropanol. Disinfection times vary from 5 to 30 min depending on the manufacturer [22]. We have found no research evaluating hydrogen peroxide as a print disinfectant.

The most hazardous consequence from dental procedures is the production of aerosols that contaminate units, handpieces, and chest fields [23]. Given that taking impression is a common practice in dentistry and due to the lack of digital flow using intraoral scanners, professionals must comply to disinfection protocols such as the use of sodium hypochlorite [24].

Agree that the high number of professionals from the survey that lack knowledge about disinfection, with 76 % not aware of protocols regarding the handling and transport of samples (impressions) and 74.6 % not following the recommended guidelines by sending to the laboratory directly, is worrisome [25,26]. With the results obtained, it can be shown that spraying with hypochlorite is the best known disinfection technique, the use of quaternary ammonium and hypochlorous acid has fallen into disuse and the use of glutaraldehyde as a disinfectant medium has not ruled out.

When evaluating current dentists, reported rates of 10.8 % (good) and 67.5 % (fair) who were not adhering to disinfection protocols, but the percentage was also high for all other professionals, further increasing the risks of cross-infections among dental care personnel, such as those in the dental laboratory [27]. Post pandemic, it was observed that 67.7 % of general dentists and 80 % of specialists were aware of the application of the disinfection protocols in the handling and transport of samples. Considering that the percentage of general dental care professionals that practice disinfection protocols is still low, it is recommended to encourage private and governmental institutions to comply in a better way.

Showed that 2 % glutaraldehyde showed a reduction of 4 log10 in 28 samples studied, demonstrating effectiveness of 10 0 %, the effectiveness of glutaraldehyde where we see that in five of the samples the effectiveness of glutaraldehyde was reduced to 0 in the number of CFU/mL, while four of them had different bacterial count [28]. When analyzing the colonies of the petri dishes, a Gram stain was performed, a sporulated microorganism was found and hence the resistance of this species to the effect of the disinfectant; the microorganism was identified as Bacillus spp [29], indicating that no disinfectant spray was effective against it [30], confirm that a combination of sodium hypochlorite and hydrogen peroxide (OX-B7) is capable of effectively killing Bacillus subtillis spores on both porous and non-porous surfaces [31]. SARS-COV2 pandemic changed deeply dental practice, increasing the attention concerning oral microbiome, antimicrobial mouthwashes and system for aerosol reduction. Future reports are needed in order to understand the complete role of SARS-COV2 in dental practice changes [32].

Stoeva et al., 2020 indicated that 43 % of health workers did not have sufficient knowledge of disinfectant substances used in printing materials, and only 50 % of clinics were applying it, noting that 181 (80.4 %) respondents used 1 % sodium hypochlorite (NaOCl) as the most common disinfectant because it maintains dimensional stability. In this study, it was determined that disinfection with hypochlorite using the 1 % spray technique is related to the use of the 4 commonly used biomaterials [[26], [33]]. Hypochlorite is effective as a disinfectant for microorganisms such as Candida albicans, S. sanguis, S. mutans, E. faecalis, E. corrodens, C. rectus, F.nucleatum, E. cloacae, K. oxytoca and K. pneumoniae; And the results of our study match this research. The Federal International Dental Association (FDI), American Dental Association (ADA), and British Dental Association (ADB) recommend a 30-min immersion in glutaraldehyde for printing materials [30], which show good results. The transfer of the sample in airtight bags is widespread among professionals [34,35]. Not many professionals use napkins soaked in disinfectant (hypochlorite) [36,37]. In the study, it is mentioned in the questionnaire that even if some impressions are sent only on napkins without a cover, these procedures should be noted to avoid subsequent complications within other health personnel, recommending on the part of the auxiliary personnel that it be handled properly, this form of transport.

Working with surveys is always complicated, the bias was limited to the maximum, however the results obtained were positive, it is recommended to redevelop a study increasing the population to other specialties, all dentists work with dental impressions.

## 5 Conclusion

The awareness of the management of dental impressions among general dentists and specialists in oral rehabilitation is limited.

## Funding

All funds used this work for publication APC were allocated by UDLA.

## 6 Informed consent statement

“Not applicable.”

## Data availability statement

Zenodo. Knowledge about methods of disinfection of dental impression among dentists from Ecuador post SARS-COV-2 pandemic. DOI 10.5281/zenodo.8417088 (https://zenodo.org/records/8417089)

## CRediT authorship contribution statement

**Myriam Lagla Abata:** Writing - original draft, Visualization, Resources, Project administration, Methodology, Investigation, Funding acquisition, Formal analysis, Data curation, Conceptualization. **Gabriela Balarezo Lasluisa:** Writing - review & editing, Writing - original draft, Supervision, Software, Resources, Project administration, Methodology, Investigation, Formal analysis, Data curation, Conceptualization. **María Rodriguez Tates:** Writing - review & editing, Writing - original draft, Formal analysis, Data curation, Conceptualization. **Byron Velásquez Ron:** Writing - review & editing, Writing - original draft, Supervision, Methodology, Formal analysis, Data curation, Conceptualization.

## Declaration of competing interest

The authors declare the following financial interests/personal relationships which may be considered as potential competing interests:Byron Velasquez Ron reports financial support and article publishing charges were provided by University of the Americas. Byron Velasquez Ron reports a relationship with University of the Americas that includes: employment.
